# Supplementary material for: Bacteria-Derived Protein Aggregates Contribute to the Disruption of Host Proteostasis
Source: Int J Mol Sci. 2022 Apr 27;23(9):4807. doi: 10.3390/ijms23094807 (PMC9103901; doi:10.3390/ijms23094807)
Supplement: Supplementary file 1 [file ijms-23-04807-s001.zip › ijms-1662214-supplementary.pdf]

Supplementary Material

Bacteria-Derived Protein Aggregates Contribute to the Disruption of Host Proteostasis

Figure S1: Bacterial utilization of butyrate as energy source. Representative bacterial growth curves in M9 minimal medium supplemented with 0.2% glucose or butyrate as the sole carbon source. Data are representative of the average of two replicates. The experiment was performed three independent times.

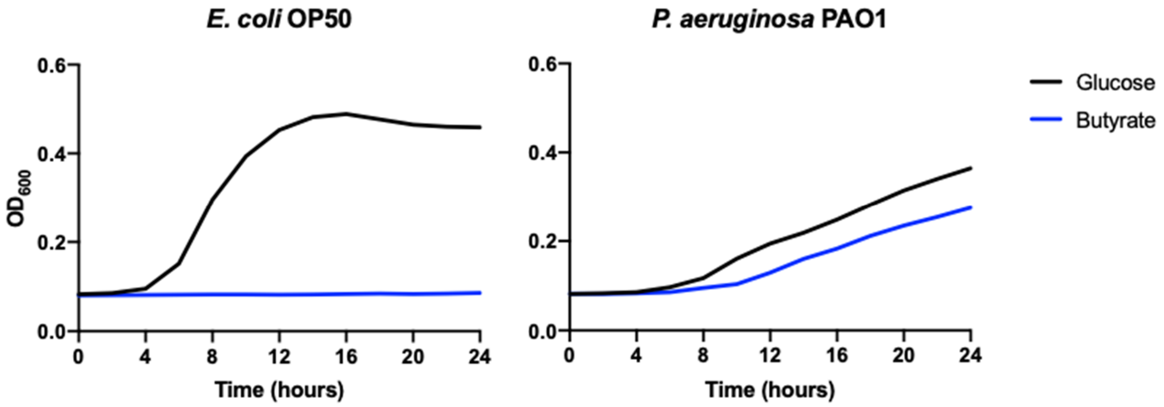

Figure S2: The effect of gentamicin on *E. coli* OP50. (**A**) Gentamicin dose-response in a plate dilution assay. Data represent three independent experiments, each in triplicates. (**B**) Overnight cultures inoculated from bacteria plated on 0 and 200 μg/mL gentamicin.

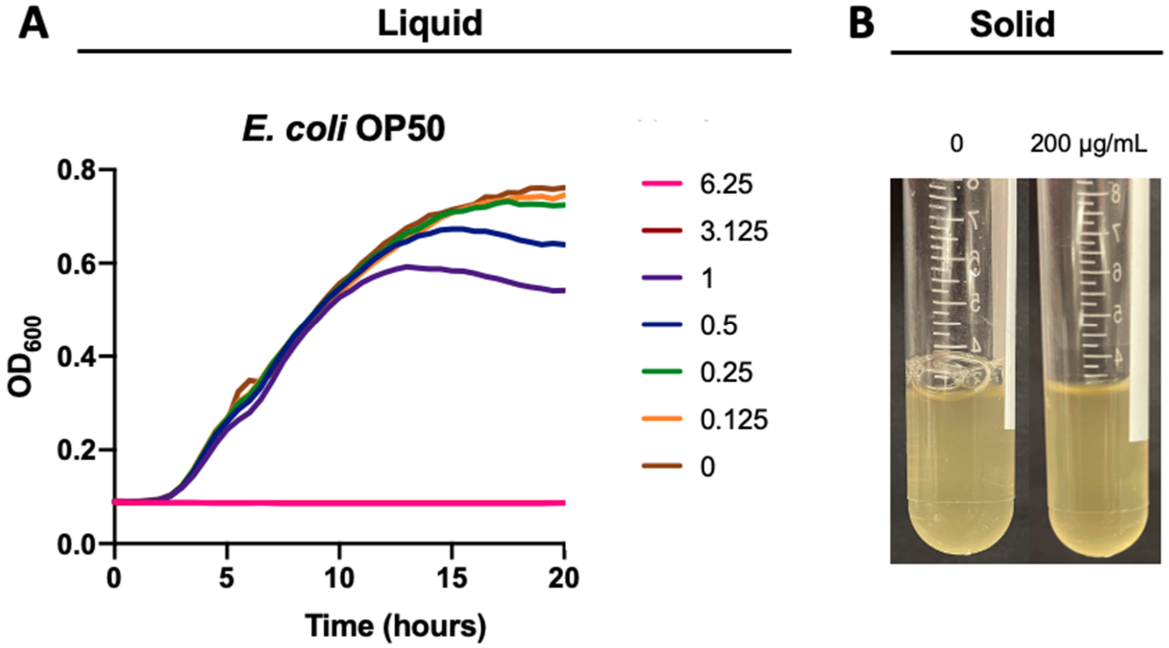

Table S1: Table of reagents and resources.

REAGENT OR RESOURCE	SOURCE	IDENTIFIER
**Bacterial strains**		
E. coli OP50	Caenorhabditis Genetics Center	WB OP50; RRID: WB-STRAIN: OP50; NCBI TaxID: 637912; DC199
Pseudomonas aeruginosa PAO1	Shuman Lab (University of Chicago)	PAO1; DC3
Prevotella corporis	BEI Resources	HM1294, MJR7716
E. coli WT	Dr. Roy Curtiss III Lab (University of Florida)	χ7122
E. coli ΔrpoS	Dr. Roy Curtiss III Lab (University of Florida)	χ7149
***C. elegans* strains**		
AM738: *rmIs297[vha-6p::q44::yfp; rol-6(su1006)]*	Morimoto Lab (Northwestern University)	AM738, Q44::YFP
**Chemicals and commercial assays**		
Cholesterol	Fisher Scientific	Cat#ICN10138201
Sodium butyrate	Fisher Scientific	Cat#A11079-22
Criterion™ XT Precast Gel	BioRad	Cat#3450124
XT 4x Sample Buffer	BioRad	Cat#1610791
XT 20X Reducing Agent	BioRad	Cat#1610792
XT MOPS	BioRad	Cat#1610788
Congo Red	Acros Organics	Cat#22962-0250
Brilliant Blue G-250	Fisher Biotech	CAS#6104-58-1
LB (Lennox)	Apex	Cat#11-125
ProteoStat™ Protein aggregation assay	Enzo	Product #ENZ-51023-KP002
Reinforced clostridial broth (RCB)	Oxoid	Code CM0149
Potassium Phosphate Monobasic	Fisher Scientific	Cat#P285-500
Ethylenediaminetetraacetic Acid (EDTA)	Fisher Scientific	Cat#S311-100
IGEPAL® CA-630	Sigma Aldrich	Cat#I8896
Brilliant Blue R-250	Fisher BioReagents	Cat#BP101-25
Isopropanol	Fisher Scientific	Cat#BP2618-1
Glacial Acetic Acid	Fisher Scientific	Cat#A38-212
Sucrose	Sigma Aldrich	Cat#S9378-500G
Tris base	Research Products International	Cat#T60040-1000.0
Acetone	Fisher Scientific	Cat#A18P-4
Trichloroacetic Acid	Fisher Scientific	Cat#A322-500
Gentamicin	Fisher Scientific	Cat#15750060
Benzonase™ nuclease	Sigma Aldrich	Cat#E1014-5KU
Lysozyme	Sigma Aldrich	Cat#L6876
Precision Plus Protein Dual Color Standards	BioRad	Cat#1610374
Phenylmethylsulfonyl fluoride (PMSF)	Sigma Aldrich	Cat#P7626
AnaeroPack™-Anaero Anaerobic Gas Generator	ThermoFisher Scientific	Cat#R681001
Fisher BioReagents™ Agar	Fisher Scientific	Cat#BP1423-2
BD Bacto™ Tryptic Soy Broth without Dextrose	Becton, Dickinson and Company	Cat#286220
Defibrinated Sheep Blood	ThermoFisher Scientific	Cat#R54020
Thermo Scientific™ AnaeroPack™ 7.0L Rectangular Jar	Fisher Scientific	Cat#23-246-387
**Equipment Used**		
Beadbug™3 Microtube Homogenizer	Benchmark Scientific	Cat#D1030
The Belly Dancer™ Orbital Platform Shaker	IBI Scientific	Cat#BDRLS0001
Tecan Infinite M Nano+ Microplate Reader	Tecan	Cat#30190087

Leica MZ10F Modular Stereo Microscope	Leica Microsystems	Cat#10450103
Zeiss Axiovert S100	Zeiss	
Archrostigmat 10X Ph1 phase-contrast infinity objective (0.25NA) Chroma EGFP/FITC long-pass filter set (19002)	Zeiss	
CoolLED pE300lite 365 dir mount STEREO	CoolLED	Cat#pE-300-LT-D-MB-YYY-ZZ
Eyepiece, 10x23B, adjustable, 3d gen	Leica Microsystems	Cat#10450910
Filter set ET GFP- MZ10F	Leica Microsystems	Cat#10450588
PowerPac 300 Electrophoresis Power Supply	BioRad	Cat#1655050
Criterion™ Cell	BioRad	Cat#1656001
Lysing Matrix A	MP Biomedicals	Cat#116910050-CF
iBright FL1000 Imaging System	Invitrogen	Cat#A32752
**Software and Algorithms**		
GraphPad Prism v9.3.1	GraphPad Software, Inc	https://www.graphpad.com
FIJI Image Processing Package	Image J	https://imagej.net/software/fiji/
BioRender	BioRender	www.biorender.com
